# Is handheld video microscopy really the future of microcirculation monitoring?

**DOI:** 10.1186/s13054-023-04642-z

**Published:** 2023-09-12

**Authors:** Matthias Jacquet-Lagrèze, Mathieu Magnin, Bernard Allaouchiche, Stanislas Abrard

**Affiliations:** 1 Service d’Anesthésie-Réanimation, Hôpital Louis Pradel, Hospices Civils de Lyon, 59, Boulevard Pinel, 69677 Bron Cedex, France; 2 Faculté de Médecine Lyon Est, Université Claude Bernard Lyon 1, 8, Avenue Rockefeller, 69373 Lyon Cedex 08, France; 3 Laboratoire CarMeN, Inserm UMR 1060, Université Claude Bernard, Lyon 1, Lyon, France; 4 Unité de Physiologie, Pharmacodynamie et Thérapeutique, VetAgro Sup, Université de Lyon, 1 Avenue Bourgelat, 69280 Marcy L’Etoile, France; 5 Pulmonary and Cardiovascular Aggression in Sepsis APCSe, UPSP 2021.A101, Université de Lyon, VetAgro Sup, Campus Vétérinaire de Lyon, 69280 Marcy L’Étoile, France; 6 Centre Hospitalier Lyon-Sud, Service de Réanimation, Hospices Civils de Lyon, 69310 Pierre-Bénite, France; 7 Service d’Anesthésie-Réanimation, Hospices Civils de Lyon, Hôpital Edouard Herriot, 5 Pl d’Arsonval, 69437 Lyon, France; 8 Institut MitoVasc, INSERM 1083 ‑ CNRS 6015, Université d’Angers, 3 Rue Roger Amsler, 49100 Angers, France

We have read with great interest the article by J. Duranteau and colleagues entitled “The future of intensive care: the study of the microcirculation will help to guide our therapies” [1]. In their review, the authors emphasize the scientific rationale for handheld video microscopy (HVM). HVM is a technique used to visualize blood flow and microcirculation in real time. The authors have described numerous advantages associated with this method. To provide the reader with a comprehensive understanding of the technical and medical issues that limit the clinical use of this technique at the bedside, we propose to highlight ten common pitfalls and drawbacks of HVM:

**1. Artifact interference**: HVM can be prone to artifacts, including motion artifacts caused by patient movement and pressure artifacts from the microscope probe. Pressure artifacts in particular are quite common and challenging, but some efforts have been made to mitigate them [2, 3]. The risk of misinterpretation of microcirculation due to pressure artifacts is of concern. These artifacts can mimic abnormal microcirculation patterns, leading to misinterpretation of the acquired images. In fact, we have observed a direct correlation between acceptable pressure artifacts and microcirculation analysis, as well as a strong association between pressure artifacts and mean arterial pressure [4].

**2. Technical complexity:** Performing sublingual video microscopy requires specialized equipment, technical expertise, and training. Analyzing the captured images and extracting meaningful data can be a complex task. Image stabilization and processing, vessel segmentation, and quantitative analysis methods may require expertise in image analysis. Analysis is a time-consuming process for an experienced operator. Automated analysis of HVM videos is reliable, but has not been validated in a prognostic assessment or interventional study. The analysis requires sufficient image quality, superior to that required by the human eye. Artifacts can lead to misinterpretation by the software and inaccurate diagnoses or conclusions. Alternatives to HVM exist: Photoplethysmography is a simpler technique that is already available in all centers and shows promising results for continuous monitoring of oxygenation and tissue perfusion [5, 6]. In contrast, HVM was able to provide continuous measurements in only 35% of patients within a short period of 3 minutes [7]. Integrated into a hemodynamic optimization protocol, tissue oxygenation monitoring varied in accordance with HVM [8, 9].

**3. Patient cooperation and invasive nature**: Obtaining clear and accurate images with sublingual video microscopy requires the patient cooperation. Excessive tongue movement or discomfort may interfere with the acquisition of high-quality images. Although sublingual video microscopy is non-invasive compared to other imaging techniques such as intravital microscopy, it still requires the placement of a device under the tongue. Some patients may find this uncomfortable or invasive, which can affect the patient compliance.

**4. Standardization and variability:** Standardizing the technique and ensuring consistency across operators or studies can be challenging. Camera settings and image acquisition protocols can vary, leading to inconsistent results and limited comparability. HVM measurements showed significant intra- and inter-observer variability [10]. Baseline microcirculatory tissue perfusion variables also vary between individuals. This substantial inter-individual variability makes it difficult to interpret single measurements and to determine individual thresholds of damage. As with several other hemodynamic variables, operators use trends in repeated measurements to monitor the evolution of HVM parameters. In comparison, a single measure of endothelial reactivity appears to provide more prognostic [11] and therapeutic information [12, 13]. In these studies, endothelial reactivity was associated with preload dependence, response to volume expansion, and outcomes in cardiac surgery.

**5. Limitations of accessible sites and depth:** The technology used in HVM can only investigate tissues covered by a thin epithelial layer. Specifically, sublingual video microscopy focuses on examining the microcirculation beneath the tongue. While it provides valuable insight into the microvasculature in this region, it may not provide comprehensive information about the microvasculature in other parts of the body, except in intraoperative settings. However, if an important physiological property can be measured at one site (e.g., sublingual), this may represent a step forward compared to not having this insight at all.

**6. Education and training:** Successful implementation of sublingual video microscopy requires trained health-care professionals who can perform the technique, interpret the images, and integrate the findings into clinical decision-making. The availability of training programs and educational resources can influence the adoption and development of the technique.

**7. Limited clinical utility**: HVM cannot directly measure tissue oxygenation. It is an indirect measure that quantifies microcirculatory perfusion and convective and diffusive oxygen transport. Other technologies have the potential to enhance the information provided by HVM regarding tissue oxygenation. However, the integration of probes for multimodal monitoring into a single device is currently unavailable. Although HVM provides valuable insights into the microcirculation, its clinical utility in guiding treatment decisions or improving patient outcomes is still under investigation. The translation of these imaging findings into actionable interventions or therapies is not yet well-established. In addition, simpler tools such as capillary refill time (CRT) have similar prognostic value [14]. CRT has the advantage of having been tested as a target in a multicenter interventional trial. It has shown promising results [11].

**8. Lack of evidence:** Demonstration of the clinical utility and effectiveness of a new technique is essential for its widespread adoption. The first randomized clinical trial of HVM did not confirm its benefit in terms of survival [15]. This study has several methodological weaknesses. The evaluation of microcirculation with an invalidated analysis method was used to guide resuscitation without linking with a clear treatment protocol. In addition, this practice was associated with an increase in the amount of fluids and vasopressors administered. Large, methodologically strong clinical trials demonstrating improved outcomes are needed to place sublingual video microscopy as a central component of patient care.

**9. Cost and availability:** Limited availability of sublingual video microscopy equipment and expertise can be a challenge in certain health-care settings or regions. The cost of the equipment and maintenance can also be a barrier to its widespread adoption. High-quality video microscopy, including the microscope, camera, lighting systems, and software, can be financially burdensome, particularly for some health-care facilities or research laboratories with limited resources.

**10. Regulatory and reimbursement challenges:** The introduction of any new medical technology involves navigating regulatory processes and securing reimbursement from health-care systems or insurance providers. These challenges can sometimes slow the adoption of new technologies, including sublingual video microscopy. Some technologies (laser Doppler and echocardiography) have the same drawback. However, it should be noted that other technologies already widely available or easily accessible are much less expensive (CRT and plethysmography). In these cases, lack of reimbursement is less of a problem.

In conclusion, HVM has significant scientific value as a tool in clinical research. However, it is important to note that HVM is not ready for routine clinical use. Manufacturers and investigators are working to improve devices, software, and clinical validation of HVM. In addition, alternative tools have emerged with interesting capabilities that are easier to use and already accessible in most centers. Therefore, while advancing HVM, it is crucial to ensure that its development does not overshadow the importance of these existing technologies and their clinical evaluation.

## Data Availability

Not applicable.
